# Analysis of 415 adverse events in dental practice in Spain from 2000 to 2010

**DOI:** 10.4317/medoral.19601

**Published:** 2014-06-01

**Authors:** Bernardo Perea-Pérez, Elena Labajo-González, Andrés Santiago-Sáez, Elena Albarrán-Juan, Alfonso Villa-Vigil

**Affiliations:** 1 Tenured Professor. School of Legal Medicine. Universidad Complutense de Madrid. Director of the School of Legal Medicine. Universidad Complutense de Madrid. Director of the Spanish Observatory for Dental Patient Safety (OESPO); 2 Contracted doctoral professor. School of Legal Medicine. Universidad Complutense de Madrid. Secretary of the Spanish Observatory for Dental Patient Safety (OESPO); 3 Associate Professor. School of Legal Medicine. Universidad Complutense de Madrid. Sub-Director of the Legal and Forensic Medical School of Madrid. School of Medicine. Universidad Complutense de Madrid. Member of the Spanish Observatory for Dental Patient Safety (OESPO); 4 Associate Professor. School of Legal Medicine. Universidad Complutense de Madrid; 5 Full Professor of Stomatology. President of Spain’s General Council of Dentists

## Abstract

Introduction: The effort to increase patient safety has become one of the main focal points of all health care professions, despite the fact that, in the field of dentistry, initiatives have come late and been less ambitious. The main objective of patient safety is to avoid preventable adverse events to the greatest extent possible and to limit the negative consequences of those which are unpreventable. Therefore, it is essential to ascertain what adverse events occur in each dental care activity in order to study them in-depth and propose measures for prevention. Objectives: To ascertain the characteristics of the adverse events which originate from dental care, to classify them in accordance with type and origin, to determine their causes and consequences, and to detect the factors which facilitated their occurrence. Material and Methods: This study includes the general data from the series of adverse dental vents of the Spanish Observatory for Dental Patient Safety (OESPO) after the study and analysis of 4,149 legal claims (both in and out of court) based on dental malpractice from the years of 2000 to 2010 in Spain. Results: Implant treatments, endodontics and oral surgery display the highest frequencies of adverse events in this series (25.5%, 20.7% and 20.4% respectively). Likewise, according to the results, up to 44.3% of the adverse events which took place were due to predictable and preventable errors and complications. Conclusions: A very significant percentage were due to foreseeable and preventable errors and complications that should not have occurred.

** Key words:**Patient safety, adverse event, medical care risk, dentistry.

## Introduction

The effort to increase patient safety has become one of the main focal points of all health care professions. We can situate the origin of this general interest in patient safety in the publishing of the study “To Err Is Human” in 1999 (To Err Is Human. Building a Safer Health System. Kohn LT, Corrigan JM, Donaldson MS), which provided troubling data on the consequences of adverse events (1). After this study, one could highlight the important initiatives by the World Health Organization, which, as of the year 2004, has led most of the initiatives in this field worldwide. Behind these efforts lie, above all, ethical but also economic reasons, as well as a desire to improve dental care quality and increase the legal security of health care professionals themselves.

In the field of dentistry, initiatives have come late and been less ambitious. However, in recent years, a significant effort has been made by the FDI World Dental Federation (FDI) and the Council of European Dentists (CED) to add to all of the other international initiatives for patient safety (2). It is important to highlight the initiative by the General Council of Odontologists and Stomatologists of Spain to create the Spanish Observatory for Dental Patient Safety (OESPO) and to promote the first “plan to prevent clinical risks in dentistry” (3).

The key concept of patient safety is that of the “adverse event.” An adverse event is any unfavorable, undesired and generally unforeseen incident caused by an error or omission during the dental treatment which has negative consequences for the patient’s health (including physical or mental damage, and/or prolonging the treatment time). These negative consequences must not be caused by the patient’s underlying disease or pathology (2).

The main objective of patient safety is to avoid preventable adverse events to the greatest extent possible and to limit the negative consequences of those which are unpreventable.

Therefore, it is essential to ascertain what adverse events occur in each dental care activity in order to study them in-depth and propose measures for prevention.

In the field of dentistry, most of the available studies are limited to descriptions of single adverse events or small series (4-13). Two broader studies were published recently, one by the National Patient Safety Agency (NPSA) (14), and another completed using surveys taken by computer amongst Finnish dentists (15). Nevertheless, all of these studies include a limited number of adverse events, with the bias inherent to the methodology used. At this time, we have no information which contains a reliable reflection of the frequency and importance of the adverse events which take place in dental practice. This information is fundamental, though. Any proposal of measures to prevent adverse events must necessarily be based on knowledge of the real situation (basically regarding frequency and severity).

In order to attempt to make up for this lack of information, the OESPO has turned to legal sources. Legal claims and court sentences tend to contain a great deal of information on the adverse events which have occurred in dental practices, as well as their causes, the circumstances surrounding each event and their consequences. On the other hand, they are significantly biased. Most adverse events with minor consequences lead to no legal claims and are therefore unknown.

This study includes the general data from the series of adverse events in dentistry of the Spanish Observatory for Dental Patient Safety (OESPO).

The objectives of this study were: to ascertain the characteristics of the adverse events which originate from dental care, to classify them in accordance with type and origin, to determine their causes and consequences, and to detect the factors which facilitated their occurrence.

## Material and Methods

This study involved the analysis of 4,149 legal claims (both in and out of court) based on dental malpractice from the years of 2000 to 2010 in Spain. The claims were taken from the Collection of court sentences on health care malpractice at the School of Legal Medicine of the Universidad Complutense de Madrid, the Archives of expert reports at this School of Legal Medicine, the Archives of patient claims of the Ethics Commission of the Official Association of Odontologists and Stomatologists of Madrid, and notices issued to the Spanish Observatory for Dental Patient Safety (OESPO) by insurance companies providing dental profession civil liability coverage.

All of the data used by the researchers were anonymous, consisting only of filiation data, in order to avoid duplication, the date and location where the adverse event took place, and the information’s source or origin.

All of the claims were studied by two researchers who worked independently. These two researchers used the same evaluation criteria when interpreting the data in the claims so that they would remain homogeneous. These researchers pre-selected all those claims which met the following requirements:

• Referring to a clearly identifiable adverse event associated with dental practice.

• Containing complete information on the location and date when the adverse event took place, with a description of the adverse event, its causes and consequences, as well as the circumstances surrounding its occurrence.

All of the pre-selected claims were then studied by “panel review,” in a panel made up of all the authors of this article, who determined that 415 claims (of the 4,149 studied) fulfilled all of the requirements to be included within the study. These 415 claims were studied jointly, and the information obtained was placed in a computerized file. The information taken from each claim which was considered included the following:

• General identification data: location and date on which the adverse event took place, and origin of the information.

• Type of adverse event ( Table 1), including the following terms:

**Table 1:**
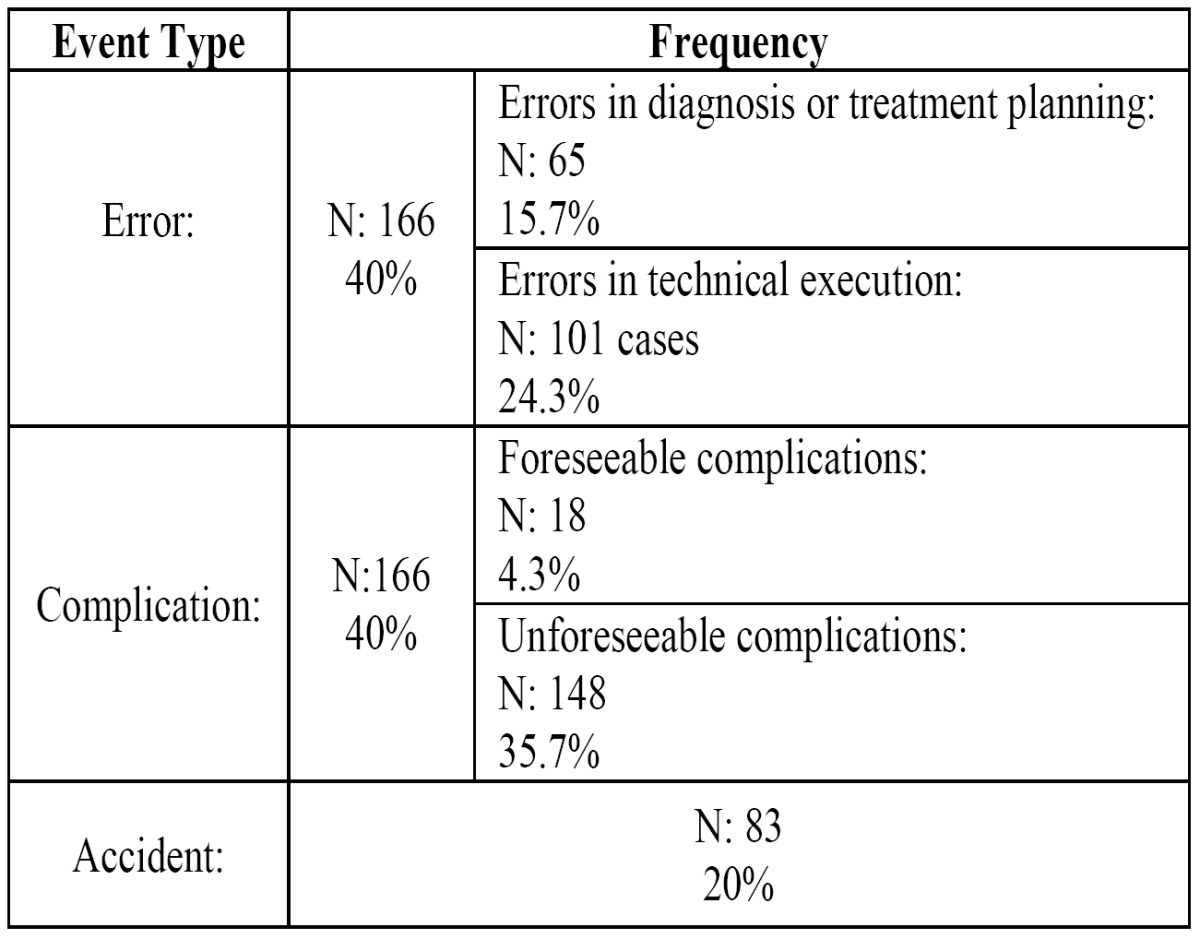
Types of adverse events which took place.

- Error: Failure in the planning, execution or patient follow-up due to a lack of aptitude or attitude of the health care professionals.

- Complication: Abnormal outcome of a process after a proper treatment.

- Accident: Unexpected and unforeseeable event which arises throughout the course of a treatment or during the patient’s stay at a health care center.

• Field of dental activity which led to the adverse event.

• Contributing factors.

• Type of health care treatment required by the patient.

• Consequences for the patient.

• Estimated degree of “preventability” (in accordance with the criteria in the available bibliography on adverse events in health care which took place in ambulatory medical care) (16).

The descriptive statistical analysis of the sample was performed by studying the frequencies of all the categories analyzed and shown on the table. Contrasting of variables was also performed using contingency tables and Pearson’s Chi-squared test of “event type” values + “field of dentistry involved,” “event type” + “damage produced” and “field of dentistry involved,” using the statistical program SPSS Statistics v19. Both the positive and negative correlations of more than n were considered: 15 cases considering a statistical correlation for values ± 2-5 and a “strong” statistical correlation for values > ± 5.

## Results

Of the 415 adverse events caused by dental treatments and included within this series, 40% of the cases were classified as “er-rors”, and a further 40% of the cases as “complications.” Classified as “accidents” were the 20% of remaining cases. The most frequent category were the “unpreventable” complications ( Table 1).

Special significance is held by some of the adverse events included in the series: 33 cases of adverse events were related with potential problems involving the instruments’ manufacturing, malfunctioning or deficient upkeep; 23 procedures were performed on the “wrong side”; there were 12 cases of swallowing dental instruments or materials; 4 cases of inhalation of dental instruments or materials; 7 events related with the prescription of drugs, and 10 cases of neuropathic pain which appeared after dental treatments.

As regards the field of dental activity which gave rise to the occurrence of the adverse event, the most frequent was “oral implantology” (25.5% of the cases), followed by “endodontics” (20.7% of the cases), and by “oral surgery” (20.2% of the cases) ( Table 2).

**Table 2:**
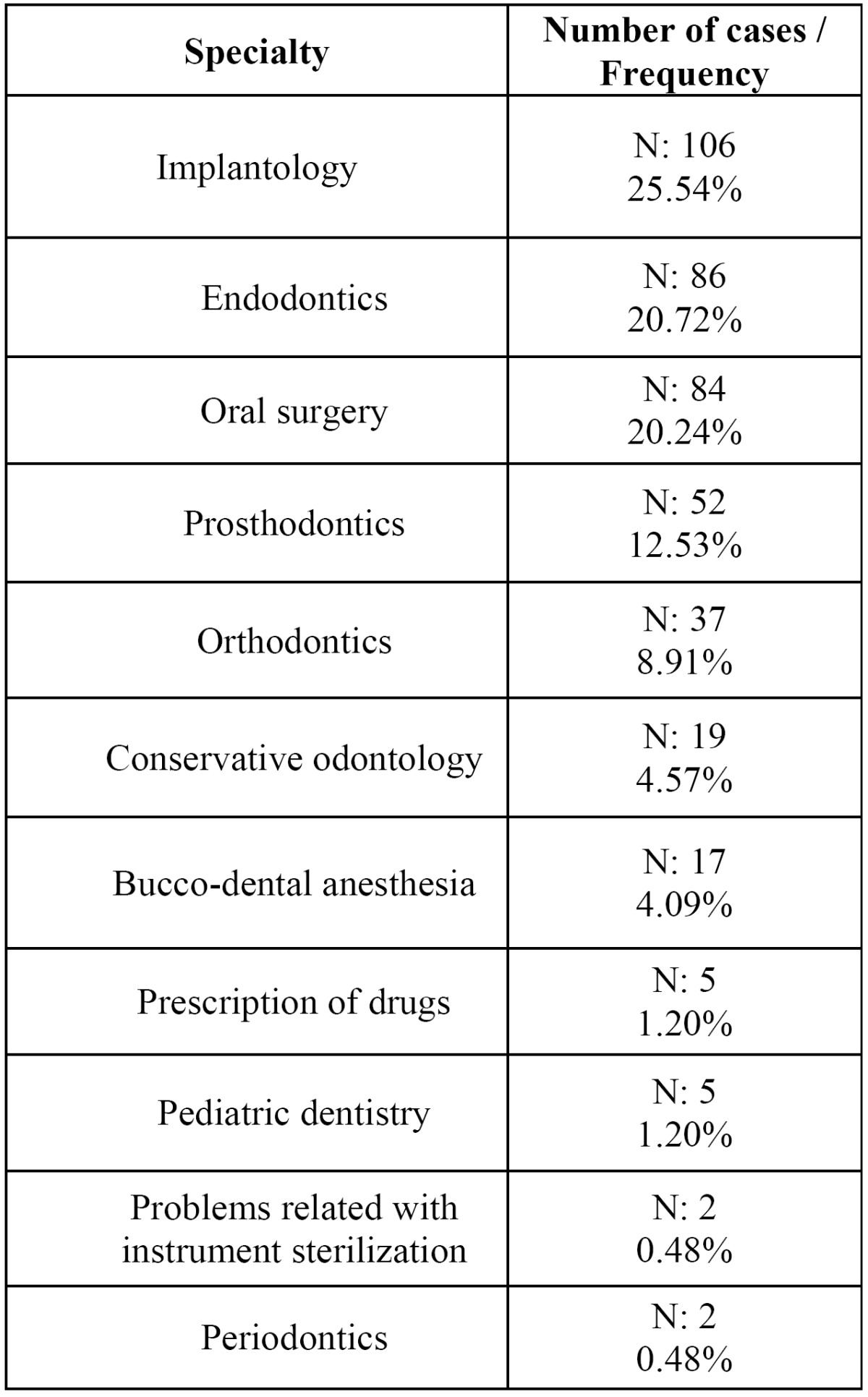
Dental specialty areas which led to the 415 adverse events studied.

In terms of the sequelae suffered by patients as a result of an adverse event, the most frequent was that of “tooth loss” (29,4% of the cases), followed by “permanent damage to nerve trunks” (18.3% of the cases), and by “significant bone loss” (10.4% of the cases) ( Table 3). The 11 deaths included in the series are very significant ( Table 4). In five cases, the existence in one patient of sequelae that can be divided into two different categories was considered.

**Table 3:**
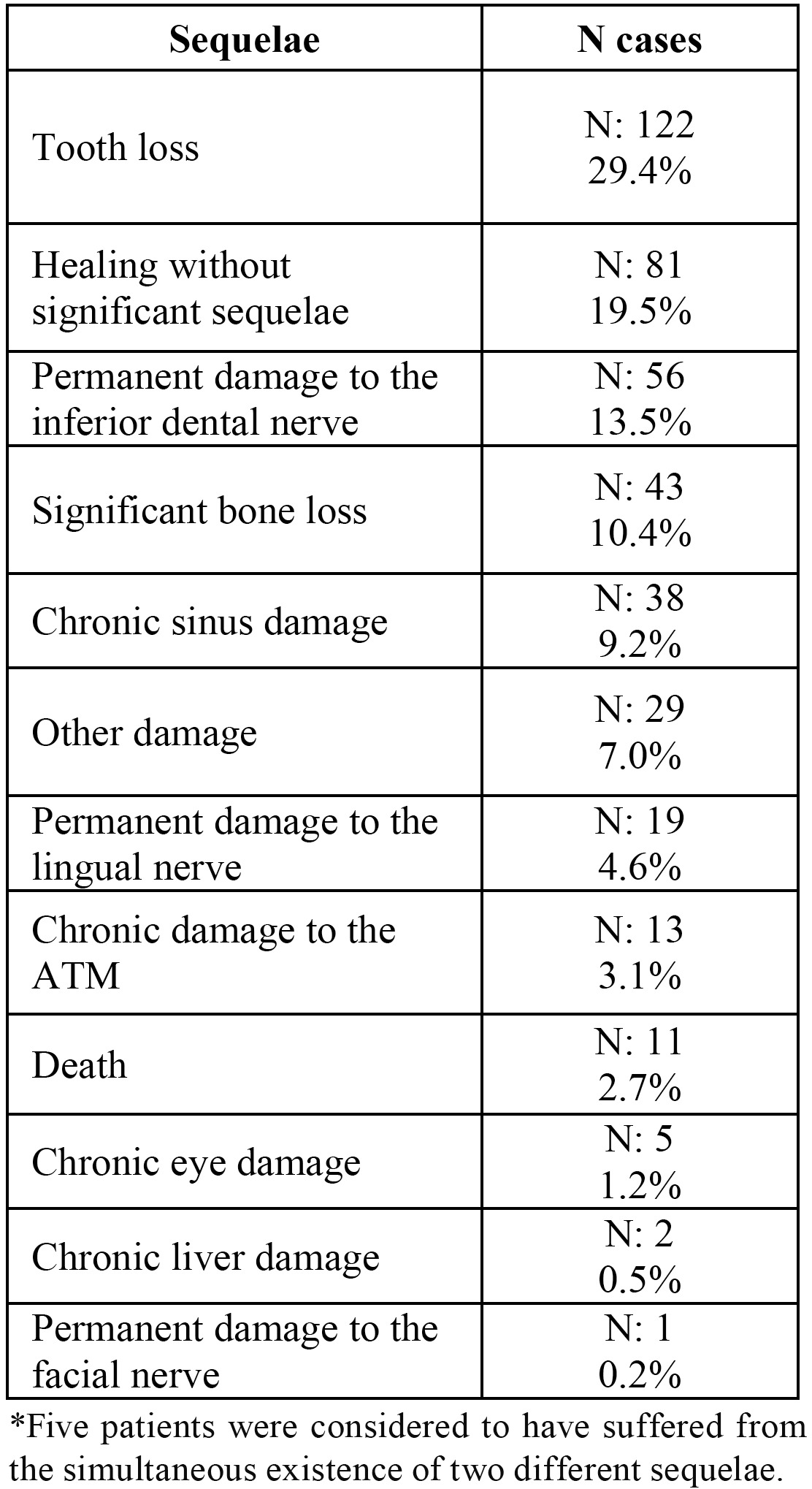
Sequelae among patients due to adverse events.

**Table 4:**
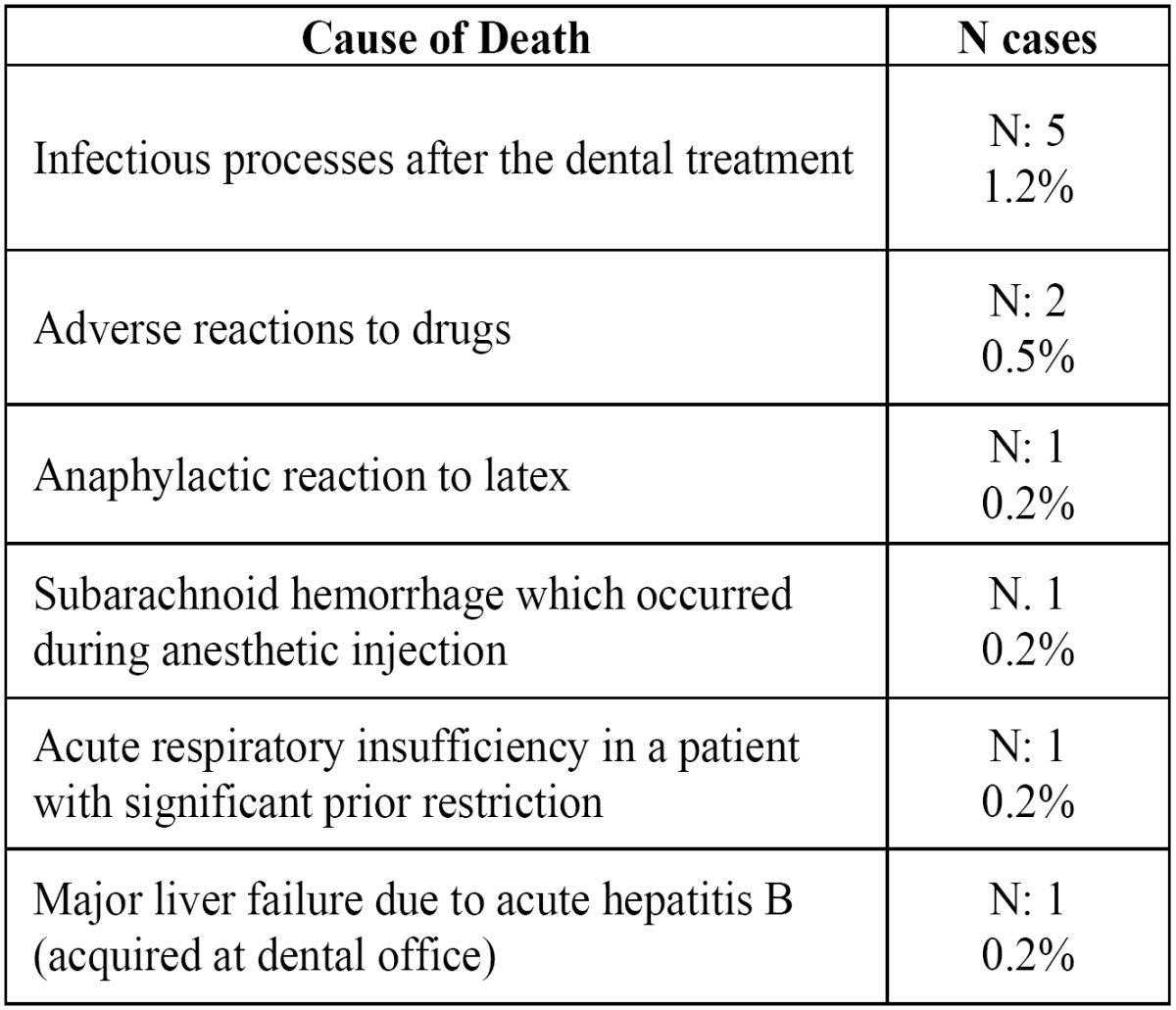
Cause of the deaths included in the series.

As for the treatment required by the patients for the treatment of the adverse event, in 46.2% of the cases “hospital admittance” was required; in 35.8% of the cases “medical treatment or hospital outpatient treatment” was required; in 18.6% of the cases “dental treatment at another center” was needed, and in 3.4% of the cases, “treatment at the very center where the adverse event took place.”

As for the estimated degree of “preventability,” the authors believe that 64.1% of the cases were “preventable,” and 35.9% of the cases were “unpreventable” despite having performed reasonable clinical practice.

The contrasting of variables was then performed using contingency tables and Pearson’s Chi-squared analysis on the values “event type” + “field of dentistry involved,” “event type” + “damage produced” and “field of dentistry involved” + “damage produced,” with a *p* value of < 0.05. Both the positive and negative correlations of more than n were considered: 15 cases considering a statistical correlation for values ± 2-5 and a “strong” statistical correlation for values.

Of the 415 adverse events which occurred, 3 were placed into two categories, because it was determined that two different adverse events had taken place as a result of one single clinical action, and therefore the number of events studied on the contingency tables is 421 cases (415+(3x2).

The following results were obtained:

a) Correlation between the “event type” and the “field of dentistry involved”:

Of the 421 cases studied, there is a positive correlation between oral surgery and errors in diagnosis, as well as between implantology and therapeutic errors. There is a strong positive correlation between surgery and unpreventable complications, between general practice and accidents, between prosthesis and therapeutic errors and between endodontics and accidents.

There is also a negative correlation between endodontics and the occurrence of non-preventable complications.

b) Correlation between the “event type” and “damage produced”: Of the 421 cases studied, there is a strong positive correlation between injuries of the lingual N. and non-preventable complications, important losses of alveolar bone and non-preventable complications, therapeutic errors and tooth losses, and accidents and healing with no significant sequelae. There is also a negative correlation between non-preventable complications and tooth losses.

c) Correlation between the “field of dentistry involved” and the “damage produced”: Of the 421 cases studied, there is a positive correlation between injuries of the inferior lingual N. (including the mandibular nerve) and oral surgery. There is also a strong positive correlation between injuries of the inferior lingual N. (including the mandibular nerve) and implantology, between im-portant losses of alveolar bone and implantology, and between prosthesis and “other damage.” 

## Discussion

The fundamental problem which exists in dental patient safety is the lack of information. Dental practice is usually dispersed and “non-structured,” being provided at small dental care centers. The professionals who undergo an adverse event at their office attempt, in the best of cases, to learn from it, but in most cases they just try to forget about it, and they almost always conceal it from other professionals or are at least reserved about the event having taken place. This means that the vast majority of information on adverse events in dentistry is lost and cannot be properly studied.

Because of this, the first consideration to take into account is the lack of representative series of adverse events caused by dental practice. The other two large series in existence (15,16) are based on data provided after the event (sometimes as much as one year) by the dentists involved. This fact must also cause bias in the data provided. The work which we are presenting herein also has an important bias: because the information comes from legal sources, we assume that the vast majority of the minor adverse events or those which were adequately solved at the center where they took place have been lost, because they did not lead to any legal claim. However, the bias found in this work also has one important advantage: the adverse events which have been included are the most serious to have taken place, and therefore they are the ones which must concern professionals the most (consider the 11 deaths included in the series). From this perspective, we believe that this series provides very useful information to professionals.

Likewise, we must bear in mind that the relevance of this legal-medical problem has a great deal to do with the likelihood that a claim will be filed. If we were to relate the population of reference or the number of dental acts performed in the interval of time studied (2000-2010) and the number of claims filed both in and out of court, then the resulting data would show an apparently low percentage, though we believe that, in order to assess the problem’s true dimension, one must take into account the exponential increase in this type of claims in recent years, thoroughly referenced in the annual reports of Professional Associations such as the General Council of Odontologists and Stomatologists.

The classification of types of adverse events which we have used (error, complication or accident) is based on the definitions proposed by the World Health Organization, with minor modifications to adapt it to our field of activity. However, differentiating amongst the different types of adverse events is occasionally difficult. For example, it is not simple to distinguish a therapeutic error from a preventable complication. The distinction has been determined by consensus among the authors based on each specific case. In our series, the number of “errors” and the number of “complications” is exactly the same (40% of the total in each case), with accidents (20%) being less frequent. The data included in the other comparable series of adverse events in dentistry do not include any distinction of these events based on their type.

As for the origin of the adverse events, in our series surgical procedures (the sum or oral surgery and implantology, 46.7%) clearly predominate over the rest. These data basically coincide with the NPSA series (14) which state that surgical specialties produced 32.8% of the total adverse events.

The treatments required by patients in order to treat adverse events are not included in the other comparable series. Our data indicate that most of the patients affected by an adverse event in dentistry (46.2%) required “hospital admittance” for their treatment. These data clearly indicate that the adverse events included in our series (which led to legal claims) were truly serious. Logic indicates to us that the vast majority of the adverse events in dentistry were treated and solved at the very center where they took place, but in our series these cases are limited to 3.4%.

The sequelae caused as a result of the adverse event are not included in the comparable studies either. And our data also reflect the “bias of severity in our series. Though in most of the cases the sequelae are limited to “tooth loss,” there are 76 cases in which permanent injuries were caused to the nerve trunks (above all to the inferior dental nerve), 38 cases of chronic sinus damage, 5 cases of permanent eye damage (two of them causing complete loss of the eyeball), and 2 cases of chronic liver damage due to viral hepatitis acquired during the dental treatment. Moreover, contrasting the variables using Pearson’s Chi-squared analysis shows that the cases of “healing without sequelae” are associated with treatments that required “hospital admittance.” Even 11 cases of death related with dental treatment or caused by other factors during the patients’ stay at the center are found in our series. Of these, some bear a direct relationship with the treatment, while others are due to prior pathologies which are manifested during treatment. All of these cases, though, have required that the dentist take active measures to treat a life-endangering emergency.

Last of all, our work studies the “preventability” of the adverse events that occurred. This data is not included in the comparable series, but it is in the series on adverse events associated with medical care in and out of hospitals. As a first consideration, we must point out the subjective nature of this conclusion. In order to attempt to alleviate this bias, this parameter was determined by a consensus of the authors based on each specific case. In our series, more than 64% of the cases were considered to be “preventable.” These data are congruent with the data on the “preventability” of the adverse events in health care which took place in ambulatory medical care (16).

## Conclusions

1. Implantology, endodontics and oral surgery treatments are those which display the highest frequencies of adverse events in this series.

2. Most of the patients affected by an adverse event in dentistry required “hospital admittance” for their treatment (46.2%). In our series, the dental adverse events treated and solved at the center itself were limited to just 3.4%.

3. The most habitual consequence of adverse events is tooth loss, though more serious sequelae have been described, such as permanent damage to nerve trunks, significant bone loss, chronic sinus damage, and even the patient’s death. In five cases, the existence of sequelae that could be placed into two categories was taken into consideration.

4. Although most of the adverse events studied involved unforeseeable complications and accidents (55.7%), a very significant percentage were due to foreseeable and preventable errors and complications that should not have occurred (44.3%).
